# Bile Leak Due to Luschka Duct Injury After Laparoscopic Cholecystectomy: A Case Report

**DOI:** 10.7759/cureus.28427

**Published:** 2022-08-26

**Authors:** Sophia Garcia, Arturo M Concepción, Cesar Wakoff

**Affiliations:** 1 General Surgery, Instituto de Pós Graduação Médica Carlos Chagas, Rio de Janeiro, BRA; 2 Bariatric Surgery, Instituto Estadual de Diabetes e Endocrinologia, Rio de Janeiro, BRA

**Keywords:** luschka duct injury, complications, biliary tree, luschka duct, laparoscopic cholecystectomy, bile leak

## Abstract

Bile leak is a common complication after laparoscopic cholecystectomy. Anatomical variations in the biliary tree can go unnoticed by the surgical team and cause complications such as this. This case report presents a patient admitted to the emergency department a week after a laparoscopic cholecystectomy due to abdominal pain and nausea. After a computed tomography, the patient was brought to the operating room for an exploratory laparoscopy, where an injured Luschka duct was found. The biliary tree has many variations that the surgeon should be aware of to minimize the risk of complications of this nature after laparoscopic cholecystectomy. There are imagining techniques with various grades of effectiveness, but in the end, the surgeon’s expertise and experience are the main factors in avoiding these complications.

## Introduction

Acute cholecystitis is one of the most prominent diseases in general surgery, accounting for over 200,000 diagnoses annually in the USA [1]. Because of this, laparoscopic cholecystectomy (LC) is one of the most frequent procedures for general surgeons. Although a relatively routinary, safe, and straightforward procedure, it still carries some difficulties, namely, anatomical variations and the severity of the pathology [2]. Biliary tract leakage is the second most frequent postoperative complication in LC and it is often due to ducts of Luschka [3]. The ducts of Luschka are rare bile ducts that directly connect the hepatic duct system to the gallbladder. These ducts appear as developmental abnormalities or anatomic variations of the bile duct system [4]. The objective of this article is to present a case of biliary leakage after an LC, and discuss what to look for to prevent this complication.

## Case presentation

A 39-year-old post-bariatric female patient without any known history of chronic illness was admitted to the emergency room seven days after undertaking a laparoscopic cholecystectomy. She was admitted due to intense abdominal pain in the right lower quadrant and two days of nausea. On physical examination, she was eupneic and hemodynamically stable. Her abdomen was flaccid, with peristalsis present, tympanic, painful at palpation on all quadrants, and without signs of peritoneal irritation. There were no other relevant findings on the rest of the physical exam. At admission, the patient’s vitals were as follows: blood pressure of 133/85 mmHg, heart rate of 70 bpm, oxygen saturation at 99%, respiratory rate of 16 bpm, and temperature of 35.6ºC. Laboratory exams are summarized in Table 1. Hemoglobin, hematocrit, and sodium were low, whereas white blood cells and C-reactive protein were high.

**Table 1:** Laboratory workup H: high; L: low.

Laboratory test	Value	Reference range
Red blood cells	3,810,000, L	4,000,000-5,200,000/µL
Hemoglobin	11.1, L	12.0-16.0 g/dL
Hematocrit	27.5, L	35.0-46.0%
White blood cells	17,200, H	4.0-11.0/µL
Bands	3	0-7%
Segmented neutrophils	78, H	40-70%
Lymphocytes	15, L	22-45%
Monocytes	4	2-10%
Platelets count	224,000	150,000-450,000/µL
Urea	33	10.0-50.0 mg/dL
Creatinine	0.7	0.6-1.3 mg/dL
Sodium	134, L	136-145 mEq/L
Potassium	4.0	3.5-5.1 mEq/L
C-reactive protein	26, H	0-0.30 mg/dL
Lactate	1.70	0.4-2.0 mmol/L

**Figure 1:**
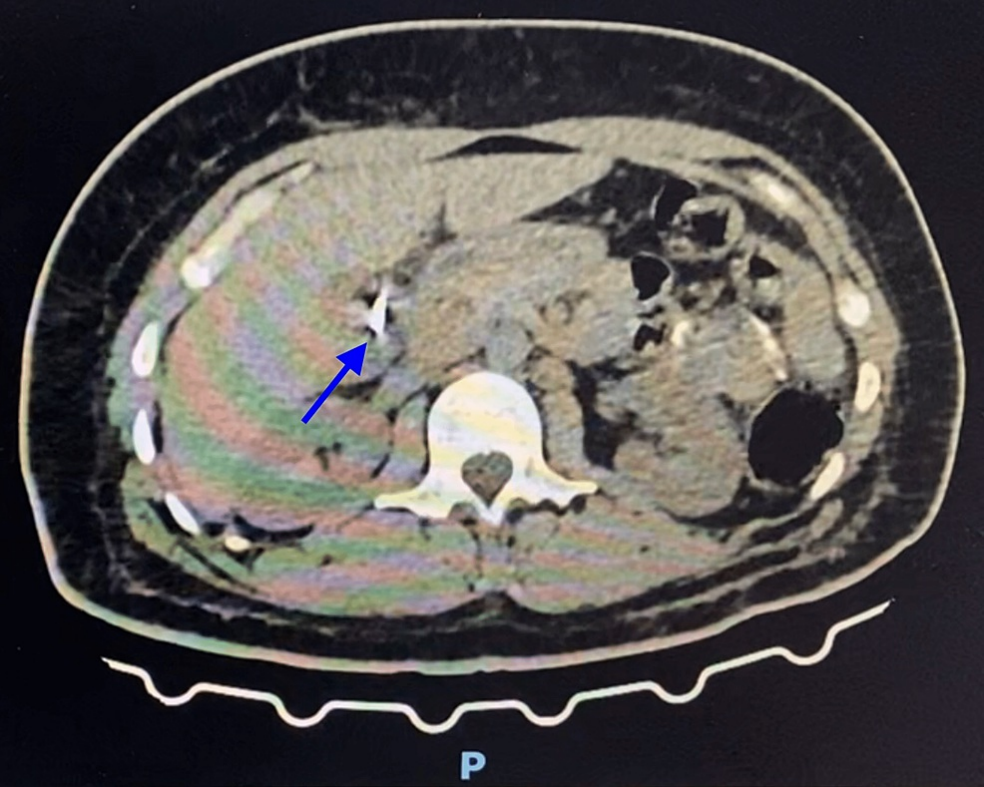
CT scan of the abdomen The image shows a small amount of liquid collected on the hepatic bed, extending medially adjacent to the VI hepatic segment (marked by the blue arrow).

**Figure 2:**
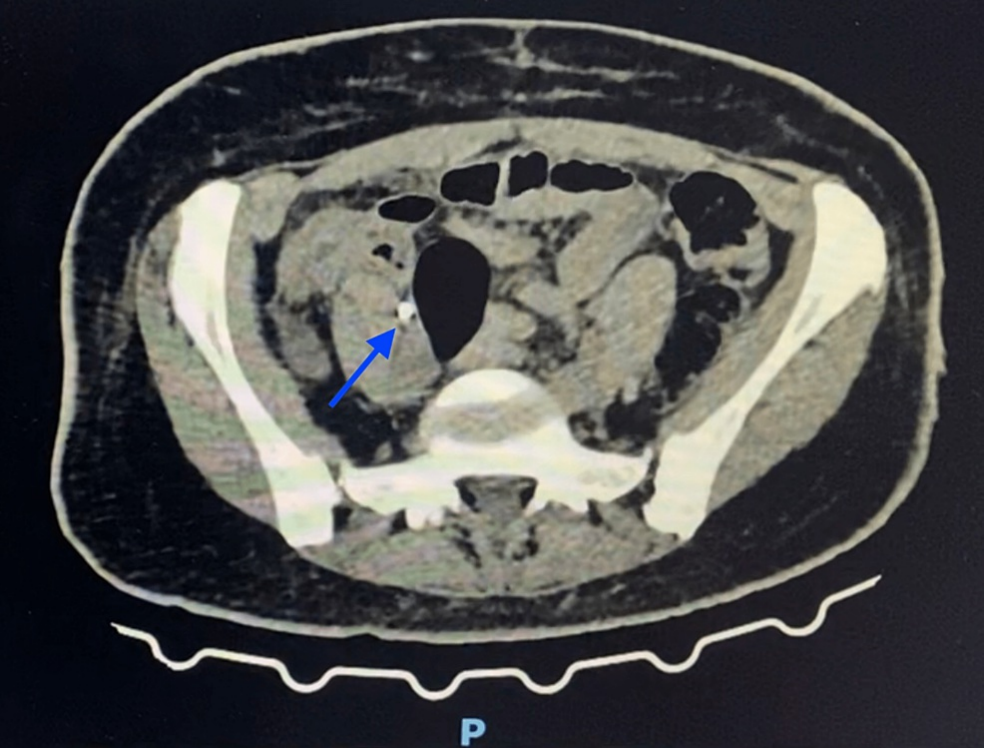
CT scan of the pelvis The image shows a moderate collection of liquid on the pelvis (marked by the blue arrow).

The patient underwent abdominal and pelvic computed tomography (CT) scan, which reported a small amount of liquid collected on the hepatic bed, extending medially adjacent to the VI hepatic segment, about 7.9 x 2.9 x 4.1 cm, which could be representing a collection in order and a small amount of liquid on the right flank, on the right paracolic gutter and in a moderate amount on the pelvis (Figures 1, 2). Based on these results, the patient was taken to the operating room for an exploratory laparoscopy.

**Figure 3:**
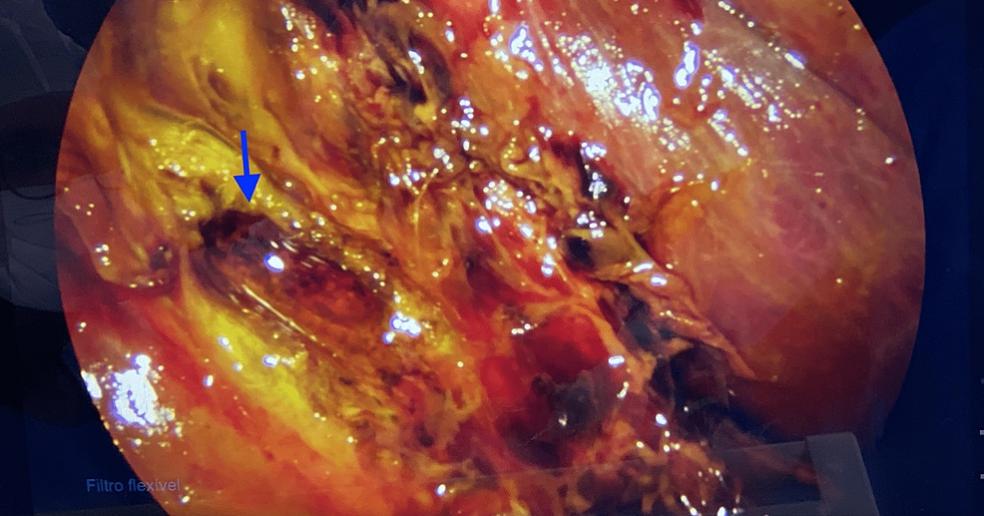
Laparoscopic view of the Luschka duct The image shows the hepatic bed with the remaining Luschka duct leaking bile (marked by the blue arrow).

**Figure 4:**
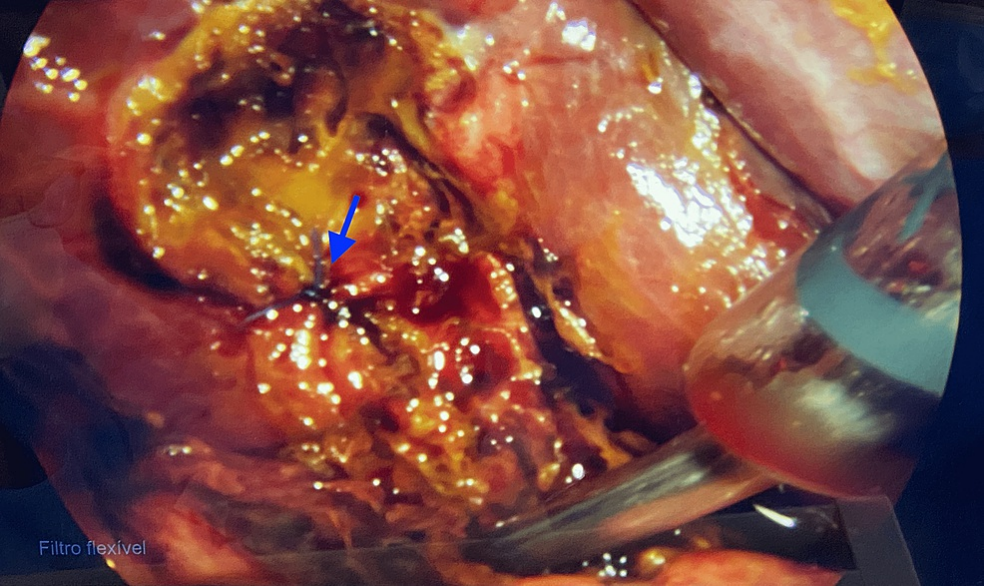
Laparoscopic view of the closed Luschka duct The image shows the Luschka duct after being closed during surgery (marked by the blue arrow).

At surgical exploration, the abdominal cavity was examined thoroughly and biliary leakage was found, flowing from a hepatic bed duct, recognized as a Luschka duct (Figure 3). The duct was closed with a 3-0 Vicryl suture (Figure 4), the abdominal cavity irrigated with warm saline, and a Blake drain left in place. The patient was prescribed ketorolac for analgesia, ondansetron for nausea relief, and ciprofloxacin and metronidazole for antibiotics during her hospital stay. Having no complications, she was discharged five days after surgery, going home with the drain. The patient had a follow-up four days later, with no complaints. At the examination, all surgical wounds were found to be healing properly and the Blake drain was removed.

## Discussion

The anatomy of the biliary tree follows the segmentation of the portal system; nevertheless, only about half of people present the “normal anatomy” [5]. It is important to know the different variants to choose the correct surgical approach and lessen the risk of complications. The most important anatomical variations to take into consideration for an LC are those of the cystic duct, such as insertion different from the middle third of the extrahepatic duct, aberrant bile ducts, and accessory bile ducts. These can result in complications like bile leakage and peritonitis. The duct of Luschka, also classified as an accessory duct or subvesical bile duct, drains the same area as the main cystic duct and its ligation does not cause any complications, but, when unnoticed, can cause significant bile leaks [6].

In 2011, Schnelldorfer et al. described four different types of subvesical bile ducts: type 1 - segmental or sectorial subvesical bile duct; type 2 - accessory subvesical bile duct; type 3 - hepaticocholecystic bile duct; type 4 - aberrant subvesical bile duct [7]. Although there are well-structured classification systems like the aforementioned, there is inconsistency in the literature regarding Luschka ducts.

To detect such variations before surgery, different imaging tools can be suggested. Ultrasonography would generally be the first one; however, it is highly operator-dependent, and thus will not always be the most effective. The second option would be tomography, which serves as confirmation for ultrasonography findings and also better evaluates the biliary tree. Unfortunately, without more invasive techniques, it is of limited use. Another important imaging technique is magnetic resonance, but its availability and cost make it infeasible as a screening tool. Endoscopic retrograde cholangiopancreatography is considered the gold standard for the identification of the biliary tree anatomy but is not cost-effective in LC [8]. Several studies have shown that intraoperative cholangiography is effective in having a clear image of the biliary tree and thus reduces the risk of bile duct injuries (BDI) [9].

However, according to the consensus made by the Research Institute Against Cancer of the Digestive System (IRCAD) Recommendations Group in 2017, there is not enough evidence to support that preoperative tests are effective in preventing surgical complications in the presence of accessory bile ducts. As a matter of fact, they concluded that not even intraoperative cholangiography is an effective tool unless there is unclear anatomy, and they emphasize the importance of the surgeon’s expertise to prevent these postsurgical complications [10].

During normal hepatobiliary development, any extra ducts stay in the liver parenchyma and become covered by liver envelope and fibrosis. The hypothesis regarding the origin of the ducts of Luschka is that these ducts do not get covered properly. Based on this conception, a careful dissection of the gallbladder, close to its wall, and a thorough examination of the fossa at the end of the surgery would help in avoiding a bile leak [11].

When detected in the intraoperative setting, repairing the defect would decrease the length and costs of hospitalization, as well as avoid the chances of infection, inflammation, or fibrosis [12]. Undetected injury of the Luschka ducts causes unspecific and minor symptoms in most cases, such as abdominal pain, nausea, vomiting, and sepsis, and may resolve on its own; however, any postsurgical symptom has to be investigated with prompt imagining to avoid further complications and identify the need of reintervention [9,13].

## Conclusions

LC is one of the most common procedures a general surgeon has to encounter. Proper patient preparation, surgical expertise, and follow-up are very important to identify anatomical variations such as Luschka ducts and therefore avoid possible complications and need for reintervention due to bile leaks or other complications.
